# Emerging concepts of shear stress in placental development and function

**DOI:** 10.1093/molehr/gaz018

**Published:** 2019-06-06

**Authors:** L C Morley, D J Beech, J J Walker, N A B Simpson

**Affiliations:** 1 Leeds Institute of Cardiovascular and Metabolic Medicine, LIGHT Laboratories, University of Leeds, UK; 2 Academic department of Obstetrics and Gynaecology, Level, Worsley Building, University of Leeds, UK

**Keywords:** placenta, shear stress, mechanosensing, Piezo1, endothelial cells

## Abstract

Blood flow, and the force it generates, is critical to placental development and function throughout pregnancy. This mechanical stimulation of cells by the friction generated from flow is called shear stress (SS) and is a fundamental determinant of vascular homeostasis, regulating remodelling and vasomotor tone. This review describes how SS is fundamental to the establishment and regulation of the blood flow through the uteroplacental and fetoplacental circulations. Amongst the most recent findings is that alongside the endothelium, embryonic stem cells and the villous trophoblast are mechanically sensitive. A complex balance of forces is required to enable effective establishment of the uteroplacental circulation, while protecting the embryo and placental villi. SS also generates flow-mediated vasodilatation through the release of endothelial nitric oxide, a process vital for adequate placental blood flow. The identification of SS sensors and the mechanisms governing how the force is converted into biochemical signals is a fast-paced area of research, with multiple cellular components under investigation. For example, the Piezo1 ion channel is mechanosensitive in a variety of tissues including the fetoplacental endothelium. Enhanced Piezo1 activity has been demonstrated in response to the Yoda1 agonist molecule, suggesting the possibility for developing tools to manipulate these channels. Whether such agents might progress to novel therapeutics to improve blood flow through the placenta requires further consideration and research.

## Introduction

**Figure 1:**
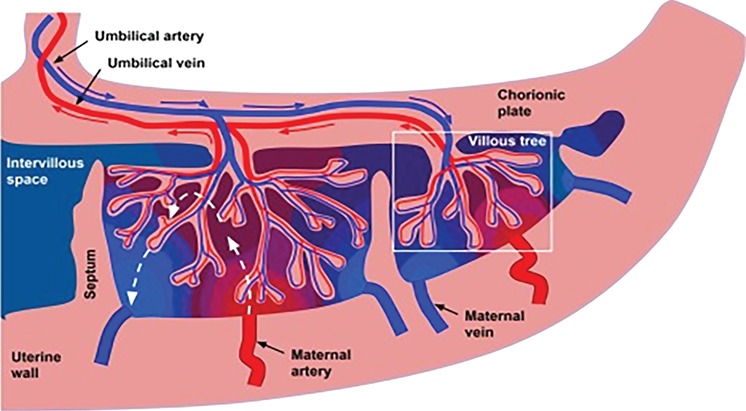
**Schematic representation of blood flow through the placenta, with contributions from the uteroplacental and fetoplacental circulations.** Blue and red arrows show the flow directions of oxygenated (red) and deoxygenated (blue) blood. The white box highlights the villous tree, comprising trophoblast cells. Vascularisation from progressive branching within the fetal circulation forms capillaries in the terminal villi, which are the functional sites of maternal-fetal exchange. Dashed white arrows demonstrate the maternal blood flow through the IVS. The red-to-blue colour gradient represents oxygenation status. Figure from Slator *et al.* (2018).

A successful pregnancy outcome is dependent upon effective placentation. This occurs within the first half of pregnancy and describes the development of two distinct but independent circulatory systems involving both the mother (uteroplacental) and baby (fetoplacental). Following implantation, extravillous trophoblast cells migrate out from placental chorionic villi and invade spiral arterioles in the maternal decidua (Kingdom, 1998). The subsequent remodelling transforms these narrow vessels into wider conduits, generating high flow and low resistance. Oxygen and nutrient-rich maternal blood is thus propelled into the evolving IVS (Fig. 1).

To support fetal circulatory and metabolic requirements, blood flow to the uterus increases progressively with advancing gestation. Maternal cardiac output is increasingly directed to the uterus secondary to falling vascular resistance and haemodynamic changes in the systemic circulation (Osol and Moore, 2014). The uterine arteries and endometrial vessels must therefore undergo vascular remodelling to accommodate these profound changes in perfusion (Park *et al.*, 2017). At the same time, the fetoplacental vasculature is developing through vasculogenesis and branching angiogenesis within the villi (Kingdom *et al.*, 2000). This arborization creates a network of capillaries enabling maximal diffusion of nutrients and gases across the maternal–fetal interface. The circulation is completed by the umbilical arteries transporting deoxygenated blood and waste to the villous tree, to enable replenishment by the maternal supply and returned to baby via the umbilical vein. Effective and responsive gas and nutrient exchange is therefore dependent upon blood flow that can adapt and vary according to circumstance (Kingdom, 1998).

A key element of vascular adaptation in pregnancy is vasodilatation through the production of nitric oxide (NO) and other agonists from the endothelial lining of blood vessels. Within the placenta, fetoplacental vessels lack autonomic innervation and control of vascular tone is therefore dependent upon these vasoactive mediators (Learmont and Poston, 1996). The most powerful physiological stimulator of NO production is shear stress (SS). This is the mechanical force generated during each cardiac cycle by the haemodynamic force of blood flow. Fluidic SS has long been recognized as being critically important for processes including angiogenesis, vasculogenesis, and control of vascular tone (Ando and Yamamoto, 2013). However, knowledge of the molecular mechanisms underpinning how cells respond to SS and how this translates into biochemical signals is only now coming to the fore. Failure of these adaptations can lead to pregnancies complicated by gestational disorders including pre-eclampsia and fetal growth restriction (FGR), the pathogenesis of which centres upon impaired spiral artery remodelling, high vascular resistance, and placental hypoperfusion, helpfully reviewed elsewhere (Kingdom, 1998). In this New Research Horizons review we present a summary of the most current knowledge of mechanosensing in both placental development and vascular function alongside the key research questions, namely as follows:

– How is SS sensed and transduced in the placenta?
– Does aberrant SS drive or contribute to placental dysfunction?
– Could identifying the molecular complexes responsible for the SS response provide novel targets for therapeutic agents to treat placental dysfunction?

Our focus is on the role of newly identified SS sensors and the opportunities this presents for future research and therapeutic applications.

## Flow dynamics and SS

**Figure 2:**
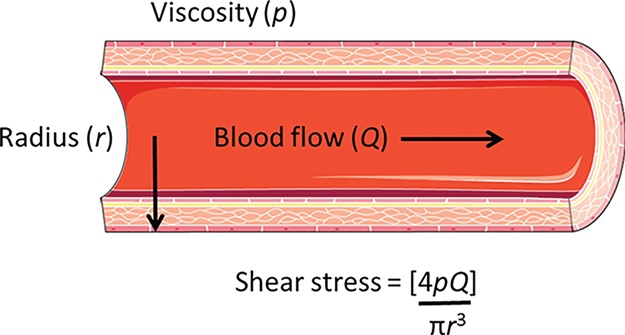
** Cross-sectional diagram of a vessel showing SS produced by blood flow.** SS in force/unit area (dyne/cm^2^) can be calculated using the equation SS = (4*pQ*)/π*r*^3^, where *r* is the vessel radius (μm), *Q* is blood flow velocity (μL/s), and *p* is blood viscosity in poise (dyne.s/cm^3^). 10 dyne/cm^2^ = 1 Pascal. Adapted from Malek *et al.*, 1999, Wareing, 2012.

SS is produced by haemodynamic force across the endothelium, and can be quantified using measurements of the inner diameter of the vessel, velocity of flow, and blood viscosity (Fig. 2) (Wareing, 2012). *In vivo* estimations of SS are complicated by the pulsatile nature of flow, the viscosity of blood contents, and the structural architecture of the vascular tree (Baratchi *et al.*, 2017). Depending on the type of vessel, its branching pattern, and variables such as temperature, flow may be laminar or turbulent and intraluminal force will therefore vary.

**Figure 3:**
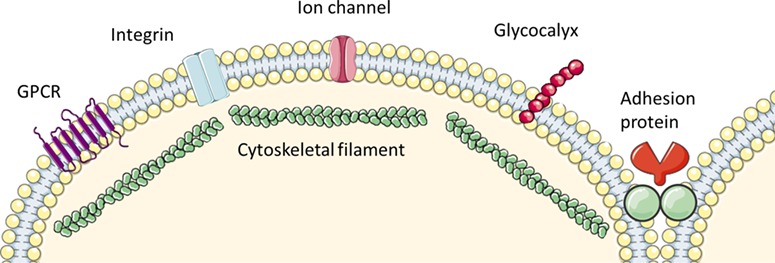
** Components of the EC, each with postulated roles in mechanosensing.** G-protein coupled receptors (GPCR) are membrane-spanning receptors e.g. GPR68 (Xu *et al.*, 2018). Integrins are membrane-spanning receptors linking cytoskeletal proteins to the cell matrix. Ion channels may be rapidly activated in response to flow. Examples include members of the TRP family and Piezo1, which are both non-selective cation channels. The glycocalyx is a matrix composed of glycoprotein and glycolipid moieties that covers and protects the cell membrane. Adhesion molecules include proteins on the cell surface (e.g. platelet adhesion cell adhesion molecule), which are expressed on cell-cell junctions. The cytoskeletal scaffold encompasses microtubules, microfilaments and intermediate protein filaments (Chatterjee, 2018). Created using SMART Servier Medical Art (LES LABORATOIRES SERVIER, SAS, France) and adapted from Shihata *et al.* (2016).

Even during embryogenesis SS is involved in the vascular expansion and remodelling required for growth. As the heart starts to beat, haemodynamic force triggers endothelial cells (ECs) to develop a vascular network through vasculogenesis and angiogenesis (Hyman *et al.*, 2017). Correspondingly, mice with a disrupted heartbeat show a lack of yolk sac vascularization alongside lethality (Huang *et al.*, 2003).

SS is sensed by the endothelium and, through mechanotransduction, activates multiple downstream signalling pathways, resulting in the release of vasoactive mediators such as NO. The endothelium is therefore dynamic, responding to local cues to control vascular homeostasis (Chatterjee, 2018). As such, the type and magnitude of SS has a significant impact on the endothelial phenotype (Malek *et al.*, 1999).

In the systemic circulation, ECs exposed to physiological SS show increased production of vasodilators and antioxidants, with a reduction in vasoconstrictors and inflammatory mediators (Malek *et al.*, 1999). Likewise, disruption of normal SS, for example at sites of turbulent flow, is considered pro-atherosclerotic (Baratchi *et al.*, 2017). Indeed, an impaired response to SS has been linked to a variety of cardiovascular disease processes such as aneurysms, thrombosis, and hypertension (Ando and Yamamoto, 2013).

Furthermore, in addition to the endothelium, mechanosensing is now being widely reported in multiple cell types that are subjected to fluid flow. This includes the epithelial villous tissue, which has important implications for placentation. It is therefore no surprise that SS and the subsequent cellular response is postulated to have multiple roles in homeostasis from acute modulation of vasomotor tone to angiogenesis and vasculogenesis (Baratchi *et al.*, 2017).

##  SS sensors

There is a growing body of literature dedicated to the identification of mechanosensors, including a variety of proteins, receptors, and transmembrane channels (Fig. 3) (Yamamoto *et al.*, 2006; Shihata *et al.*, 2016; Chatterjee, 2018). The architecture of the cell itself may also be involved in the SS response. The cytoskeleton is composed of protein filaments that scaffold the ECs and may be deformed by shear on the cell surface. This impacts on cellular components, such as integrins (membrane spanning receptors), focal adhesion proteins, and the extracellular matrix, where the force is transduced (Chatterjee, 2018). Integrins themselves may also sense SS, affecting the cytoskeletal filaments directly (Baratchi *et al.*, 2017; Chatterjee, 2018). Force may also be transmitted to the cytoskeleton via the heparan sulfate, chondroitin sulfate, and hyaluronic acid moieties of the glycocalyx on the EC membrane. As such, deformation of the cytoskeleton by shear may result in EC reorganization and remodelling (Chatterjee, 2018).

It is possible that these putative factors are co-dependent with several SS sensors working in conjunction to form a mechanosome complex (Chatterjee, 2018). One such complex is proposed by Tzima *et al.* (2005). Here the authors suggest that platelet EC adhesion molecule (PECAM-1/CD31), which is expressed on endothelial cell-cell junctions is the direct SS sensor. This results in activation of the junction receptor vascular endothelial cadherin, alongside vascular endothelial growth factor (VEGF) receptor 2 (Tzima *et al.*, 2005). These events may trigger activation of the integrins (Tzima *et al.*, 2005).

The identification of specific fast-acting mechanosensors, which could be pharmacologically manipulated, is of particular interest. For example, the transient receptor potential (TRP) channels are a major class of calcium ion (Ca^2+^)-permeable and non-selective cationic channel. Ca^2+^ influx through TRP channels produces smooth muscle contractility, alterations in vascular permeability, and remodelling (Baratchi *et al.*, 2017). However, in murine models knock out of the TRP channel, TRPV4, did not affect survival, while TRPP2 homozygous knock-out mice had delayed lethality (Hartmannsgruber *et al.*, 2007; Garcia-Gonzalez *et al.*, 2010).

The ion channel subunit Piezo1 was first discovered in 2010 (Hyman *et al.*, 2017). Since then this mechanosensitive membrane protein has risen to prominence as a major player in SS sensing and is considered in more detail below.

### Piezo1 proteins

**Figure 4:**
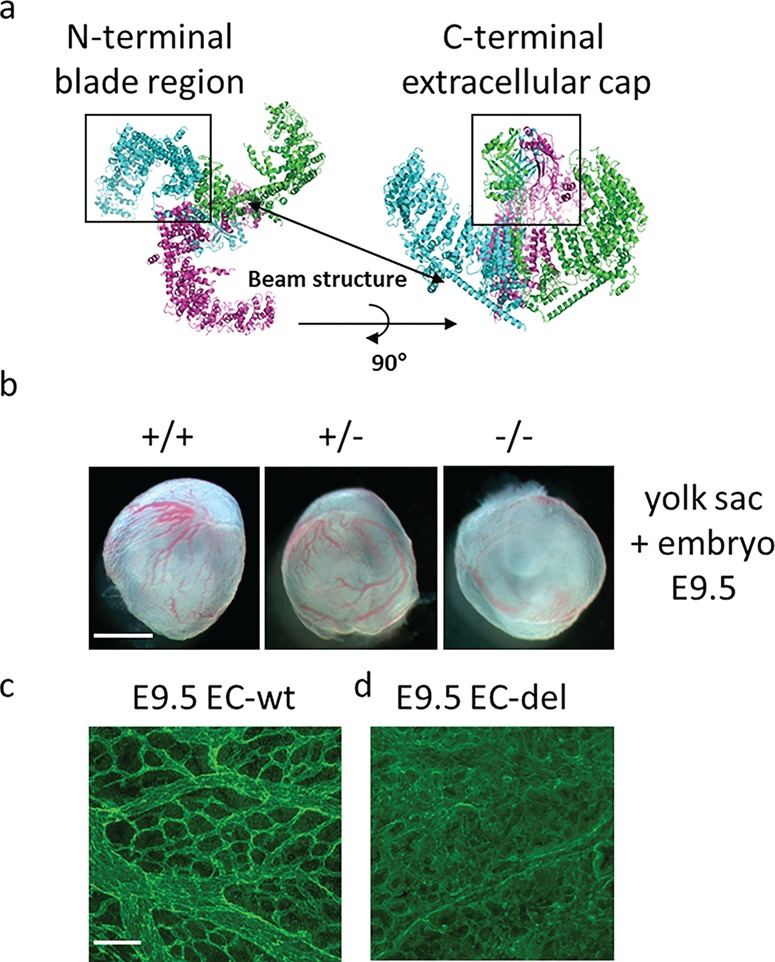
** Structure and importance of Piezo1 for murine vascular development.** (**a**) Cryo-electron microscopy Piezo1 trimeric structure produced using PyMol (Schrodinger Inc. Cambridge, UK) using PDB code 5Z10. Each monomer is represented as ribbons (monomer 1 cyan, monomer 2 magenta, monomer 3 green) (Zhao *et al.*, 2018). (**b**) Images of sibling yolk sacs (containing embryos) at E 9.5. Scale bar, 1 mm. Piezo1 wild-type (++), haplotype (+/−), and homozygous knock out (−/−) are depicted (Li *et al.*, 2014). (**c** and **d**) Dissected yolk sacs stained for CD31, showing wild-type (c) and endothelial-specific *Piezo1*-modified (d) samples. EC: endothelial cell. Both sets of images show reduced vascularization at mid-gestation (Li *et al.*, 2014).

Piezo1 channels are distinct from conventional ion channels due to their size (900 kDa), structural complexity as a large trimer of Piezo1 proteins, and rapid activation/inactivation (Chatterjee, 2018). Cryo-electron microscopy has revealed the structure of Piezo1 to be propeller-like, with three highly flexible blades and a central cap enclosing a Ca^2+^-permeable non-selective cation channel (Fig. 4a and b) (Guo and MacKinnon, 2017; Saotome *et al.*, 2018). The extracellular blade components are thought to act as force sensors within the vessel lumen, regulating the gated channel (Guo and MacKinnon, 2017). The most recent evidence suggests that these channels directly sense and modulate membrane tension via mechanisms, which are currently unknown (Cox *et al.*, 2016; Syeda *et al.*, 2016).

Our group reported that Piezo1 responds to SS in human umbilical vein ECs (HUVECs) (Li *et al.*, 2014). We then identified downstream proteins affected by Piezo1 in these HUVECs, cultured in both static and flow conditions. After Piezo1 depletion using siRNA there was a reduction in endothelial NO synthase (eNOS) activation and furthermore, abolition of VEGF-evoked phosphorylation of eNOS at serine 1177—known to enhance eNOS activity (Li *et al.*, 2014). The mechanism by which SS-activated Piezo1 leads to NO release is still under investigation. Wang et al. (2016) suggest that Piezo1 is required for the release of endothelial ATP. This leads to activation of the G protein coupled receptor P2Y2 and downstream NO production (Wang *et al.*, 2016). This suggests that Piezo1 has a key role in regulating vasodilation through NO.

To further investigate the significance of Piezo1, our lab generated a mouse model with a disrupted endogenous *Piezo1* gene (Li *et al.*, 2014). Inheritance of both the global homozygous and endothelial-specific knock out was lethal in pups at mid-gestation [embryonic days (E) 9.5–11.5]. The phenotype we observed was reduced yolk sac vascularization and FGR prior to *in utero* demise (Fig. 4b). Closer inspection revealed initial endothelial lattice formation preservation, but angiogenesis was significantly inhibited (Li *et al.*, 2014) (Fig. 4c). The finding that Piezo1 appears critical for early murine vascular development was also demonstrated by another group, suggesting that Piezo1 could be a major player in human embryonic and placental vascular development (Ranade *et al.*, 2014).

In humans, autosomal recessive loss of function mutations in *PIEZO1* causes generalized lymphatic dysplasia (Fotiou *et al.*, 2015, Lukacs *et al.*, 2015). This can present prenatally as non-immune fetal hydrops, which can result in death or pulmonary compromise at birth, secondary to pleural effusions (Martin-Almedina *et al.*, 2018). Oedema can also recur in childhood and be generalized, affecting the face, limbs, and genitals (Martin-Almedina *et al.*, 2018). The most well-known *PIEZO1* mutation is autosomal dominant, where gain of function results in dehydrated xerocytosis, a disorder of erythrocyte stability (Zarychanski *et al.*, 2012). Interestingly, this can also present with perinatal oedema (Martin-Almedina *et al.*, 2018). The similarity between both phenotypes suggests that Piezo1 has a critical role in sensing and developing the lympho-vascular flow system, although this is very much an emerging area of work (Hyman *et al.*, 2017).

Research into Piezo1 in other areas of adult disease is rapidly expanding. Recent publications include findings in the pancreas, gastrointestinal tract epithelium, cardiomyocytes, and chondrocytes (Alcaino *et al.*, 2017; Liang *et al.*, 2017; Servin-Vences *et al.*, 2017; Romac *et al.*, 2018). Despite these studies highlighting the pathological relevance of Piezo1, its role in mechanosensing in the placenta remains under researched.

##  SS sensing in pregnancy

### Preimplantation

From the moment of conception, a preimplantation embryo is subjected to the force of fluid flow as it traverses the uterus by peristalsis. To determine if embryos can sense SS, Xie et *al.,* (2006) exposed mouse embryos to either shear or static conditions (Xie *et al.*, 2006). They found no surviving embryos after 24 h of shear at 1.2 dyn/cm^2^ and reduced blastocyst cell numbers. An increase in apoptotic markers and stress-activated protein kinase [mitogen-activated protein kinase ( MAPK)] suggested that shear was transduced prior to lethality (Xie *et al.*, 2006). Shear-induced lethality after removal of the zona pellucida (ZP) suggests that the ZP may protect the embryo from mechanical force. This has implications for embryo handling in assisted conception transfer procedures, although transient SS caused by repeated pipetting did not affect development, despite increased MAPK phosphorylation (Xie *et al.*, 2007).

New research has identified Piezo1 channels in murine embryonic stem cells (Del Marmol *et al.*, 2018). This raises the question of whether Piezo1 activation by SS is important for cell differentiation. The lack of lethality in the knock out mouse models until mid-gestation suggests that Piezo1 is not critical for early development but may have fine-tuning roles that are not yet fully identified (Del Marmol *et al.*, 2018).

### Post implantation

Transfer of oxygen to the fetus is critically dependent on flow past, and effective diffusion across, the trophoblast (Kingdom, 1998). Despite the importance of SS, extravillous trophoblast cells also form plugs within the spiral arteries, effectively preventing the flow of maternal blood into the IVS (James *et al.*, 2018). It is well known that a hypoxic environment is important for early development. For example, in cases of miscarriage, erythrocytes have been found prematurely in the IVS (Jauniaux *et al.*, 2000). Alongside regulation of oxygen tension, the presence of plugs will also impact on haemodynamics. Until recently, it was thought that plugs prevented any blood from entering the IVS until the end of the first trimester. However, recent work has suggested that capillary-sized channels enable a small, constant influx of blood that increases dramatically after 12 weeks of gestation (Roberts *et al.*, 2017). Nevertheless, low SS conditions of <2 dyne/cm^2^ (<0.2 Pa) are produced in the IVS (James *et al.*, 2018). James *et al.* (2012) found that at very low fluidic shear (0.5 and 2 dyne/cm^2^) trophoblasts did not undergo migration. However, at increased SS (4 and 6 dyne/cm^2^) migration in the direction of flow occurred. This suggests that high SS at this early stage would damage vascular remodelling by stimulation of migration away from the site of invasion (James *et al.*, 2012). Low SS may therefore be protective of delicate villous tissue through prevention of physical and oxidative stress. Breakdown of plugs coincides with the end of the first trimester, where increasing blood flow generates SS which encourages trophoblast migration (James *et al.*, 2018).

Placental villi protruding from the trophoblast interact with maternal blood bathing the IVS. Scanning electron microscopy has been used to demonstrate minimal villous growth in BeWo cells and cultured human villous trophoblast under static conditions. By contrast, when exposed to SS, villi formation started at 1 hour. Miura *et al.* (2015) found short villous projections at high SS but these protrusions were longer (>2 μm) at low SS, increasing over 12 hours. If, however, SS was stopped, the villi decreased (Miura *et al.*, 2015). This therefore suggests that placental villi sense and respond to flow in their fluidic environment. Furthermore, there is a minimum requirement for SS as a “critical external cue” for villous formation (Miura *et al.*, 2015).

When investigating the mechanism behind SS-induced villi formation, Miura *et al.* (2015) found that application of SS to BeWo cells increased intracellular calcium concentration (Ca^2+^). Correspondingly, culturing cells in the presence of a Ca^2+^-chelator inhibited villous growth (Miura *et al.*, 2015). They suggest that the Ca^2+^ channel TRPV6, a member of the TRP family, is a candidate mechanosensor here (Fig. 3). Silencing TRPV6 using siRNA resulted in loss of the SS-induced intracellular Ca^2+^ response. They hypothesize that activation of TRPV6 results in re-localization of Ezrin (a protein linking the membrane and cytoskeleton) within the cell. Rapid Ezrin phosphorylation, but a lack of change in gene expression in response to SS, suggests that trophoblast cells are hyper-responsive to their dynamic fluid environment.

This interplay between the uteroplacental and fetoplacental circulations occurs throughout pregnancy, whereby SS produced by the flow of maternal blood in the IVS continues to affect the villous trophoblast. VEGFs produced by the placenta, including placental growth factor (PlGF), are contributors to angiogenesis and vasodilatation in the placenta. Exposing trophoblast cells from term placentas to SS at 1 dyne/cm^2^ resulted in increased PlGF secretion and intracellular Ca^2+^ influx (Lecarpentier *et al.*, 2016a). Magnetic resonance imaging of term pregnancies has revealed maternal blood flow velocity of 0.94 (+/− 0.14 mm.s^−1^), which has been used to estimate SS values of <5 dyne/cm^2^ in the IVS (Lecarpentier *et al.*, 2016b). This computer modelling suggests that at term, maintaining low SS at the maternal-fetal interface remains important, and may promote maximal exchange of nutrients and waste.

### Regulation of fetoplacental blood flow

Increased synthesis of NO, alongside other vasodilators such as prostacyclin, has been demonstrated in a perfused human placental cotyledon model (Wieczorek *et al.*, 1995). Flow-mediated vasodilatation has also been shown in the perfused placenta model, and in chorionic plate arteries with incremental increases in flow decreasing vascular resistance (Learmont and Poston, 1996; Jones *et al.*, 2015). Increased eNOS expression was also found in ovine fetoplacental ECs (FpECs) after exposure to SS (Li *et al.*, 2004). Correspondingly, NO inhibition increased vascular resistance, a finding which was substantially elevated in FGR samples. Likewise, human placental arterial ECs from the chorionic plate demonstrated increased NO in response to SS (Jones *et al.*, 2015). Wire myography of chorionic plate arteries showed relaxation in response to the NO donor sodium nitroprusside (Mills *et al.*, 2005). Correspondingly, the authors observed increased relaxation in FGR samples (Mills *et al.*, 2005).

This suggests that vessels from dysfunctioning placentae have the capacity to vasodilate over-and-above those from a healthy pregnancy.

In FGR, the anatomy of the placental vascular tree is altered with smaller numbers of immature villi, suggestive of impaired branching angiogenesis. This occurs alongside endothelial dysfunction and creates a hypoxic environment (Kingdom *et al.*, 2000). As such, subsequent increases in transmural pressure in small vessels will heighten placental vascular tone, generating greater SS forces (Krause *et al.*, 2013). This is supported by the previously mentioned findings of reduced flow-mediated dilatation in FGR (Jones *et al.*, 2015). In addition, total eNOS expression is higher in cells cultured from FGR samples (Myatt *et al.*, 1997; Jones *et al.*, 2015). We propose that mechanotransduction has an important role in regulating placental vascular tone under normal conditions and when SS is elevated in FGR. As such, heightened SS leading to increased production of NO by ECs may act in a compensatory capacity to overcome vascular resistance. When endothelial dysfunction is severe enough to prevent the normal response to SS, mechanosensory compensation will be insufficient and FGR may worsen (Jones *et al.*, 2015; Morley *et al.*, 2018).

A challenge to our hypothesis includes the finding that SS increases PlGF in trophoblast cells in a dose-dependent manner (Lecarpentier *et al.*, 2016a). There are possible considerations:

– As Lecarpentier *et al.*, (2016a) suggest, maternal placental hypoperfusion in FGR may instead ‘decrease’ the SS exerted on the trophoblast. This would result in the low levels of PlGF associated with FGR and pre-eclampsia (Lecarpentier *et al.*, 2016a).
– The failure of PlGF production may represent trophoblast dysfunction and failed compensation, rather than being indicative of the SS response.
– There are microsites with different levels of shear and it is not surprising to find differing responses from the trophoblast versus those observed in uterine, endometrial and FpECs. This is supported by Sprague *et al.* (2010) who discussed the differences in viscosity secondary to the arrangement of erythrocytes, between capillaries and larger capacity vessels (Sprague *et al.*, 2010).

#### Piezo1 in the fetoplacental circulation.

‘Stretch-activated non-selective cation channels’ were described in HUVECs in 1998 and it was found that they were involved in Ca^2+^ influx (Kohler *et al.*, 1998; Brakemeier *et al.*, 2002). A doubling in density of these channels was found in HUVECs from pregnancies affected by pre-eclampsia, although their molecular identity remained unknown (Kohler *et al.*, 1998). Given our finding that Piezo1 is present in HUVECs and is critical for vascular development in the murine embryo, we sought to establish if Piezo1 had a role in human FpECs.

**Figure 5:**
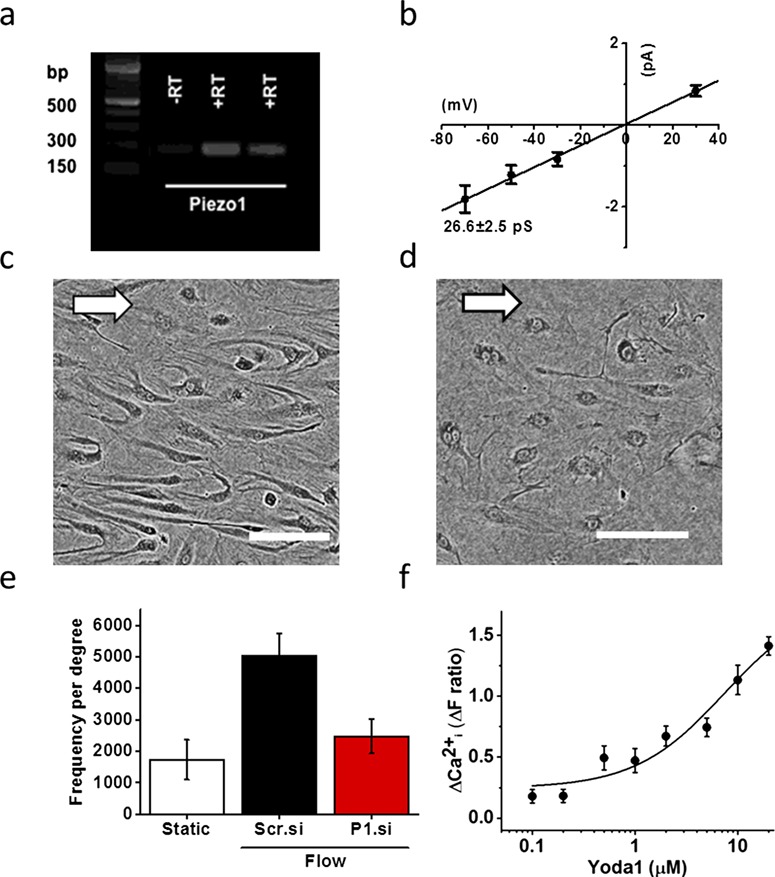
**Piezo1 is present in placental endothelium and is required for the SS response.** (**a**) Quantitative PCR performed on mRNA isolated from FpECs readily detected *PIEZO1* mRNA expression. (**b**) Patch clamp recordings from freshly isolated placental arterial ECs showed constitutive Piezo1 channel activity (26 pS). (**c**) FpECs transfected with scrambled siRNA (Scr.si) after exposure to 48 h of SS caused by an orbital shaker showed alignment (arrow depicts direction of flow, scale 100 μm). (**d**) Lack of alignment in FpECs exposed to SS after Piezo1 depletion with siRNA (P1.si) (scale 100 μm). (**e**) Quantification of orientation analysis showing significantly reduced alignment after P1.si versus Scr.si transfection (*P* < 0.05); mean height (SEM) after Scr.si 5674.611 (760.763), P1.si 2185.42 (548.786), static 1728.812 (632.188) (*n* = 3/*N* = 2). transfection (*P* < 0.05). (**f**) Changes in intracellular Ca^2+^ occurred in response to increasing concentrations of Yoda1 compared with vehicle control (mean ± SEM; mean responses to Yoda1 fitted with the Hill equation suggested an approximate EC_50_ of 5.36 μM). Adapted from Morley *et al.* (2018).

Our data revealed consistent Piezo1 gene expression in FpECs, alongside flow-dependent Piezo1 channel activity (26 pS) in fresh arterial ECs extracted from the chorionic plate (Fig. 5a and b). When Piezo1 was depleted in FpECs using siRNA, alignment in response to flow was significantly diminished, suggesting the importance of Piezo1 for SS sensing (Fig. 5c–e). It remains to be determined how SS activates Piezo1 channels in FpECs but our data support the findings in murine endothelium, suggesting that these channels are activated by SS in a membrane-delimited manner (i.e. in excised cell-free membrane patches) (Rode *et al.*, 2017).

### Regulation of uteroplacental blood flow

The production of endothelial NO, alongside prostacyclin and endothelial hyperpolarizing factor, has long been recognized as essential for the vasodilatory component of the expansive remodelling in the uteroplacental circulation (Osol and Moore, 2014). This can be demonstrated in uterine arteries, where an 8-fold increase in eNOS activity was observed in normal pregnancy (Nelson *et al.*, 2000). This has also been shown in animal models where eNOS inhibition prevented uterine artery remodelling (Ko *et al.*, 2018). The augmentation of NO in pregnancy may be secondary to SS generated by the increase in blood flow through the uterine vasculature. This is in conjunction with growth factors such as VEGF and PlGF, and endocrine signals (Osol and Moore, 2014). Successful vascular adaptation normalizes the SS, although definitive values for SS in human uterine vessels both in normal pregnancy and in gestational disorders remains unreported.

The response of uterine artery ECs to SS can be demonstrated by their alignment in the direction of flow (10–20 dyn/cm^2^, based on an assumption of physiological arterial SS in the systemic circulation of 6–40 dyn/cm^2^) (dela Paz *et al.*, 2012; Park *et al.*, 2017). Alongside the morphological change in response to SS, Park *et al*. (2017) showed increased expression of VEGF receptor-3 in human uterine artery ECs. This receptor is thought to be involved in angiogenesis and its expression in response to fluidic shear occurred independently of its ligand. Although alterations in flow through the uterine vessels may contribute to vascular remodelling, the mechanisms transducing SS into the expression of growth factors remains unknown. Piezo1 has been detected in the uterine artery endothelium of the rat (John *et al.*, 2018). It is therefore possible that Piezo1 activation may lead to NO production, alongside expressions of VEGFs, but further work is required to confirm this.

**Figure 6:**
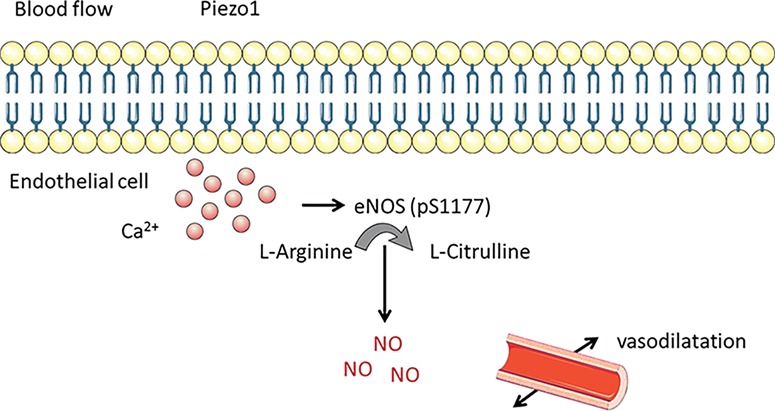
**Schematic illustrating the possible mechanism by which SS activation of Piezo1 results in vasodilatation through the production of NO by phosphorylation of eNOS at serine site 1177.** NO: nitric oxide, p: phosphorylation, eNOS: endothelial NO synthase. Created using SMART Servier Medical Art (LES LABORATOIRES SERVIER, SAS, France).

A low-sodium diet was used by Bigonnesse *et al.* (2018) to generate a rat model of FGR characterized by reduced uteroplacental perfusion. Impaired blood flow velocity and increased resistance occurred secondary to reduced uterine vessel diameters. In these hypoperfused placentae, increased NO activity was observed, leading the authors to conclude that enhanced production was compensating for vasoconstriction (Bigonnesse *et al.*, 2018). This feeds into our hypothesis that alterations in SS are detected by mechanosensors leading to the release of NO as a compensatory mechanism (Fig. 6).

Analysis of placental bed biopsies showed that increasing intraluminal flow led to dilatation of the small myometrial resistance arteries. By contrast, in women with pre-eclampsia, the SS response was blunted, with absent flow-mediated dilatation (Kublickiene *et al.*, 2000). In contrast, inhibition of eNOS increased myogenic- and norepinephrine-induced tone in arteries from both controls and pre-eclampsia (Kublickiene *et al.*, 2000). As such, NO may still have a role in regulation of vascular tone in pre-eclampsia, but the *SS-*mediated NO release appears to be absent. Failure of SS-induced vasodilatation may therefore contribute to impaired uteroplacental blood flow (Kublickiene *et al.*, 2000; Osol *et al.*, 2017).

## Therapeutic applications

In 2015 a synthetic agonist of Piezo1 was discovered, called Yoda1 (Syeda *et al.*, 2015). This compound activates Piezo1 prolonging the opening of the channel, leading to downstream effects via influx of Ca^2+^ and other cations into the cell.

This exciting step allows us to manipulate Piezo1 function chemically. In our FpECs, Yoda1 caused strong elevation of the intracellular Ca^2+^ concentration with a 50% effect occurring at about 5.4 μM (Morley *et al.*, 2018) (Fig. 5f). Depletion of Piezo1 using siRNA suppressed the Yoda1 response, consistent with it being mediated by Piezo1 channels (Morley *et al.*, 2018). Similar findings have also been demonstrated in iatrogenic pancreatitis secondary to high pressure generated surgically. This showed that Piezo1 channels present in the murine pancreas could be stimulated by Yoda1 (Romac *et al.*, 2018). These findings suggest that the response to mechanical force can indeed be manipulated using drugs.

Although Yoda1 lacks the pharmacological properties of a drug for human use, this is a first step towards developing new compounds with better pharmacokinetic properties. Most recently, a new set of Piezo1 chemical activators (Jedi) has been developed. Interestingly, activation by Jedi required the force-sensing architectural components of the Piezo1 channel to be present to be effective (Wang *et al.*, 2018). Likewise, new inhibitors are also being developed by medicinal chemists, such as Dooku1, which counteracts the effects of Yoda1 (Evans *et al.*, 2018).

## Future research

The precise nature of SS and the role of mechanosensors at different stages of placental development still need to be determined. Accurately representing *in vivo* SS conditions in the *in vitro* setting remains a challenge. Sprague *et al.* (2010) have described the SS variations between vascular beds, type and size of vessel, and even at each bifurcation. It is unlikely that results obtained from pure laminar SS exposure are accurately representative in this context (Sprague *et al.*, 2010). This was recently highlighted where both laminar and disturbed flow were found to activate the same initial pathways involving Piezo1, P2Y2, and G protein receptors. However, only disturbed flow resulted in downstream activation of integrins and focal adhesion proteins (Albarran-Juarez *et al.*, 2018).

To overcome this, Sprague *et al.* (2010) suggest using mathematical models and computer simulation to better reflect the complexity of SS and improve the congruency of current experimental models with *in vivo* vasculature. Future studies may also use microfluidic chambers to set targeted shear rates across cell co-cultures, such as placental trophoblast and endothelium (Gnecco *et al.*, 2017).

The impact of *PIEZO1* mutations on the lymphovascular system supports the importance of the channel for human development (Martin-Almedina *et al.*, 2018). However, as far as we are aware, no studies have investigated Piezo1 in the first trimester. Our work has shed new light on mechanosensing in FpECs from healthy term pregnancies (Morley *et al.*, 2018). Urgent work is therefore required to establish differences in SS sensing and mechanosensor expression between normal endothelium and that from pre-eclampsia and FGR. Likewise, the role of specific mechanosensors in the trophoblast remains unexplored. Our working hypothesis links Piezo1 activation by fluid flow to downstream production of NO (Fig. 6). However, it is possible that other extracellular or intracellular proteins may interact with and/or regulate Piezo1 channels and this remains to be determined. The availability of new compounds such as Yoda1, Jedi, and Dooku1, which specifically target the Piezo1 channel, offers opportunities for further fundamental research into this important mechanosensor. The potential for, and consequences of, these mechanosensor modulators crossing the placenta and their effect on the baby remains to be determined. For example, we do not yet know if such agents would have vascular bed specificity within uteroplacental and/or fetoplacental circulations, nor the effect this would have on maternal and fetal wellbeing given the importance of maintaining a balanced pressure gradient between the two circulations.

## Conclusion

Previous studies, alongside our own work, have shown that uterine arteries, FpECs, and trophoblast cells are mechanically sensitive. The body of literature describing the role of SS sensing and subsequent signalling in multiple vascular functions within the placenta is growing. It is becoming apparent that SS has implications for a developing pregnancy, from establishing the conceptus through to adequacy of placental blood supply at term. The role of SS appears to be nuanced however, depending upon gestation and potentially, cell type. This paradox stems from the threshold required for survival while preventing damage to the embryo and villi. As pregnancy progresses however, profound vasodilation and adaptation to flow are required to enable adequate placental perfusion. As such, a delicate balance in fluid forces is required for the complex processes of embryo establishment, uteroplacental remodelling, and fetoplacental vascular development. This therefore requires tight control by regulators capable of sensing and transducing force at each stage of development.

Until relatively recently, the molecular mechanisms of mechanosensing have been unknown. Accelerated research has led to the discovery of Piezo1, which is functionally active in FpECs. We are far from piecing together how Piezo1 activation ultimately results in the complex and orchestrated processes of vascular remodelling. However, it is striking that even *in vitro* Piezo1 activation is necessary for alignment of ECs to SS.

SS mechanotransduction and subsequent signalling, as well as the interplay between these mediators, and clinical and genetic risk factors for placental dysfunction need to be studied for a better understanding of placental haemodynamic regulation. The ability to manipulate mechanosensory complexes and influence maternal-fetal blood flow with new compounds could pave the way for new treatments to prevent the consequences of placental dysfunction.
